# Crystal structure, Hirshfeld surface analysis and density functional theory study of 6-methyl-2-[(5-methyl­isoxazol-3-yl)meth­yl]-1*H*-benzimidazole

**DOI:** 10.1107/S2056989021002723

**Published:** 2021-03-19

**Authors:** Ahlam Idrissi, Karim Chkirate, Nadeem Abad, Bahia Djerrari, Redouane Achour, El Mokhtar Essassi, Luc Van Meervelt

**Affiliations:** a Laboratory of Heterocyclic Organic Chemistry URAC 21, Pharmacochemistry Competence Center, Av. Ibn Battouta, BP 1014, Faculty of Sciences, Mohammed V University, Rabat, Morocco; b Department of Biochemistry, Faculty of Education & Science, Al-Baydha University, Yemen; c KU Leuven, Chemistry Department, Celestijnenlaan 200F box 2404, Leuven, (Heverlee), B-3001, Belgium

**Keywords:** crystal structure, density functional theory, benzimidazole, hydrogen bond, Hirshfeld surface analysis

## Abstract

In the title mol­ecule, C_13_H_13_N_3_O, the isoxazole ring is inclined to the benzimidazole ring at a dihedral angle of 69.28 (14)°. In the crystal, N—H⋯N hydrogen bonds between neighboring benzimidazole rings form chains along the *a-*axis direction. Hirshfeld surface analysis indicates that the most important contributions to the crystal packing are from H⋯H (48.8%), H⋯C/C⋯H (20.9%) and H⋯N/N⋯H (19.3%) inter­actions. The optimized structure calculated using density functional theory at the B3LYP/6–311 G(d,p) level is compared with the experimentally determined structure in the solid state. The calculated highest occupied mol­ecular orbital (HOMO) and lowest unoccupied mol­ecular orbital (LUMO) energy gap is 4.9266 eV.

## Abstract 2

The isoxazolyl-benzimidazole moiety is not planar. In the crystal, N—H⋯N hydrogen bonds between neighboring benzimidazole rings form chains along the *a*-axis direction.

## Chemical context   

Nitro­gen-based structures have attracted increased attention in structural and inorganic chemistry in recent years because of their inter­esting properties (Lahmidi *et al.*, 2018 ▸; Chkirate *et al.*, 2020*a* ▸; Taia *et al.*, 2020 ▸; Al Ati *et al.*, 2021 ▸). The benzimidazole family, particularly compounds containing the 2-methyl benzimidazole moiety, is important in medicinal chemistry because of their wide range of pharmacological applications including as anti­microbial and anti­tubercular agents (Ranjith *et al.*, 2013 ▸), potential urease enzyme inhibitors (Menteşe *et al.*, 2019 ▸) and anti­bacterial agents (Chkirate *et al.*, 2020*b* ▸). In particular, isoxazolyl benzimidazole derivatives are used as analgesic and anti-inflammatory agents (Kankala *et al.*, 2013 ▸). They are also potent and orally bioavailable bromo­domain BET inhibitors (Sperandio *et al.*, 2019 ▸). Given the wide range of therapeutic applications for such compounds, and in a continuation of the work already carried out on the synthesis of compounds resulting from 1,5-benzodiazepine (Chkirate *et al.*, 2001 ▸, 2018 ▸, 2019*a* ▸,*b* ▸,*c* ▸, 2021 ▸), a similar approach gave the title compound, 6-methyl-2-[(5-methyl­isoxazol-3-yl)meth­yl]-1*H*-benzimidazole C_13_H_13_N_3_O (I).
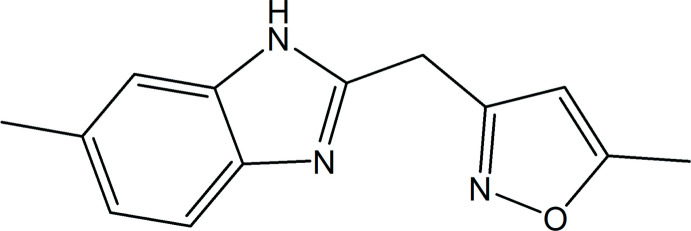


Besides the synthesis, we also report the mol­ecular and crystal structures along with the results of a Hirshfeld surface analysis and density functional theory computational calculations carried out at the B3LYP/6– 311 G(d,p) level.

## Structural commentary   

**Figure 1:**
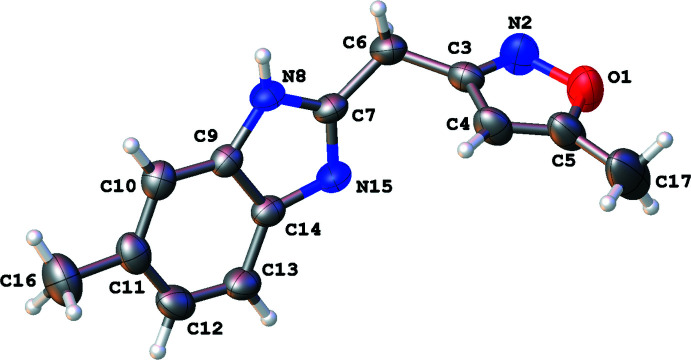
Mol­ecular structure of the title mol­ecule with the atom labeling scheme and 50% probability ellipsoids.

The title compound crystallizes in the ortho­rhom­bic space group *Pbca* with one mol­ecule in the asymmetric unit (Fig. 1 ▸). The mol­ecule is not planar, as indicated by the torsion angles C4—C3—C6—C7 [−40.4 (4)°] and C3—C6—C7—N15 [−46.0 (4)°]. The best plane of the isoxazole ring (O1/N2/C3–C5; r.m.s. deviation = 0.003 Å) makes a dihedral angle of 69.28 (14)° with the best plane of the benzimidazole ring (C7/N8/C9–C14/N15; r.m.s. deviation = 0.015 Å). Both methyl groups are in the same plane as the ring to which they are attached [deviation of C17 from the isoxazole plane = 0.016 (6) Å, deviation of C16 from the benzimidazole ring = 0.067 (4) Å].

## Supra­molecular features   

**Figure 2:**
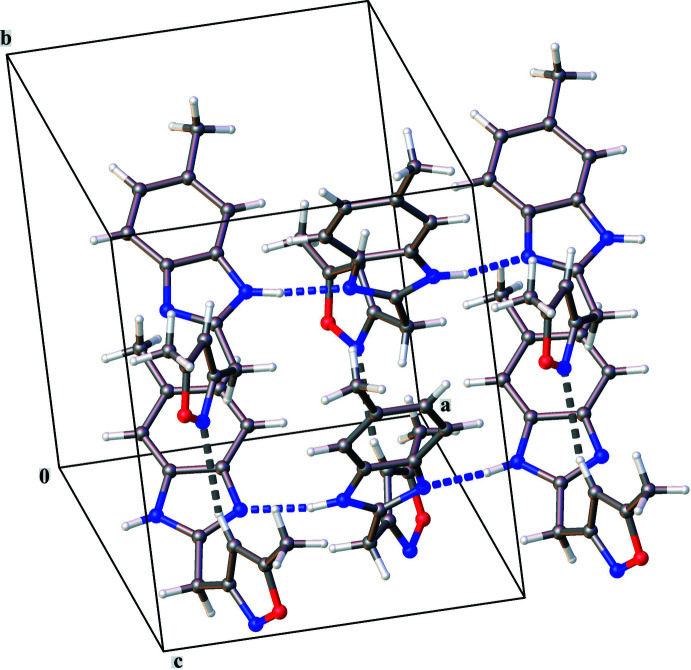
Partial crystal packing of the title compound. N—H⋯N hydrogen bonds are shown by blue dashed lines and C—H⋯N hydrogen bonds by gray dashed lines.

The crystal packing is characterized by N—H⋯N and C—H⋯N inter­actions (Fig. 2 ▸, Table 1 ▸). Chains of mol­ecules running in the *a*-axis direction are formed by N8—H8⋯N15^i^ hydrogen bonds between neighboring benzimidazole rings [symmetry code: (i) −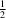 + *x*, *y*, 3/2 – *z*]. Parallel chains inter­act through C4—H4⋯N2^ii^ hydrogen bonds between neighboring isoxazole rings [symmetry code: (ii) 3/2 – *x*, 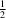 + *y*, *z*] resulting in the three-dimensional structure. The crystal packing contains no voids.

## Hirshfeld surface analysis   

**Figure 3:**
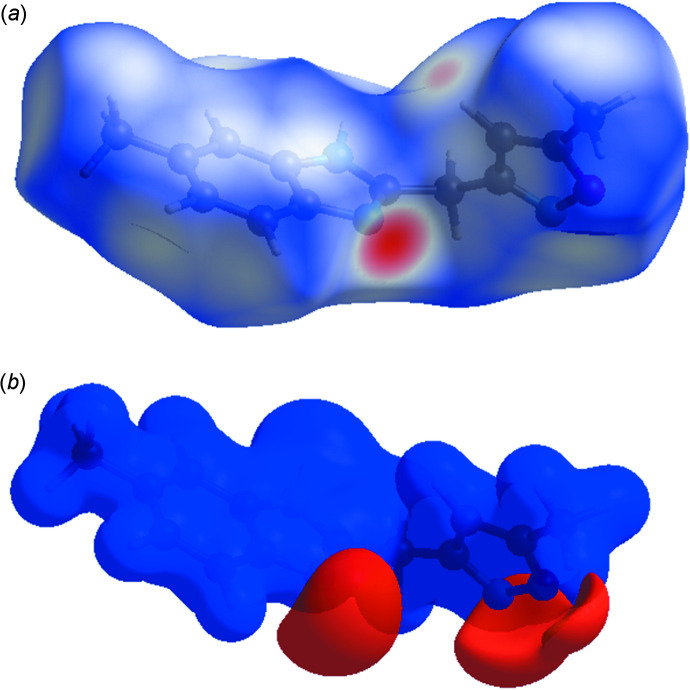
(*a*) View of the three-dimensional Hirshfeld surface of the title compound, plotted over *d*_norm_ in the range −0.6149 to 1.3177 a.u. (*b*) View of the three-dimensional Hirshfeld surface of the title compound plotted over electrostatic potential energy in the range −0.0500 to 0.0500 a.u. using the STO-3 G basis set at the Hartree–Fock level of theory.

**Figure 4:**
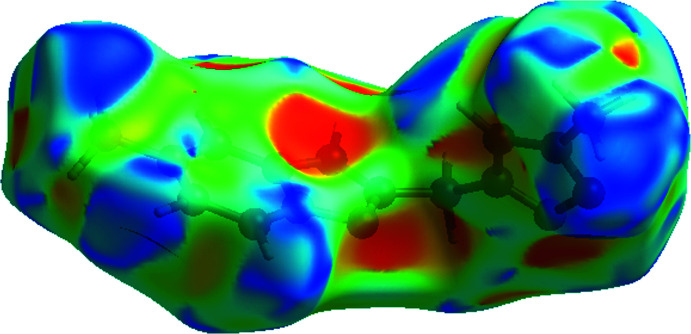
Hirshfeld surface of the title compound plotted over shape-index.

The *CrystalExplorer* program (Turner *et al.*, 2017 ▸) was used to investigate and visualize the inter­molecular inter­actions of (I). The Hirshfeld surface plotted over *d*_norm_ in the range −0.61 49 to 1.3177 a.u. is shown in Fig. 3 ▸ *a*. The red spots are close contacts with a negative *d*_norm_ value and represent N—H⋯N and C—H⋯N inter­actions. The white regions representing contacts equal to the van der Waals separation and a *d*_norm_ value of zero are indicative of the H⋯H inter­actions. The electrostatic potential using the STO-3G basis set at the Hartree–Fock level of theory and mapped on the Hirshfeld surface over the range ± 0.05 a.u. clearly shows the positions of close inter­molecular contacts in the compound (Fig. 3 ▸ *b*). The positive electrostatic potential (blue region) over the surface indicates hydrogen-donor potential, whereas the hydrogen-bond acceptors are represented by negative electrostatic potential (red region). The shape-index (Fig. 4 ▸) generated in the ranges −1 to 1 Å reveals that there are no significant π–π inter­actions (normally indicated by adjacent red and blue triangles).

**Figure 5:**
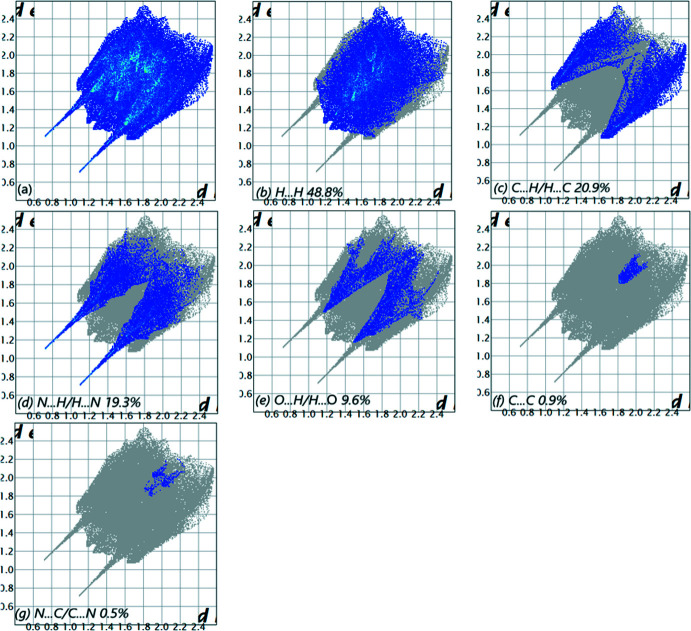
The full two-dimensional fingerprint plots for the title compound, showing (*a*) all inter­actions, and delineated into (*b*) H⋯H, (*c*) H⋯C/C⋯H, (*d*) H⋯N/N⋯H, (*e*) H⋯O/O⋯H, (*f*) C⋯C and (*g*) C⋯N/N⋯C inter­actions. The *d*_i_ and *d*_e_ values are the closest inter­nal and external distances (in Å) from given points on the Hirshfeld surface.

The overall two-dimensional fingerprint plot (McKinnon *et al.*, 2007 ▸) is shown in Fig. 5 ▸ *a*, while those delineated into H⋯H, H⋯C/C⋯H, H⋯N/N⋯H, H⋯O/O⋯H, C⋯C and C⋯N/N⋯C contacts are illustrated in Fig. 5 ▸ *b*–*g*, respectively, together with their relative contributions to the Hirshfeld surface (HS). The most important inter­action is H⋯H, contributing 48.8% to the overall crystal packing, which is reflected in Fig. 5 ▸ *b* as widely scattered points of high density due to the large hydrogen content of the mol­ecule, with the tip at *d*_e_ = *d*_i_ = 1.28 Å. In the presence of C—H inter­actions, the pair of characteristic wings in the fingerprint plot delineated into H⋯C/C⋯H contacts (20.9% contribution to the HS), Fig. 5 ▸ *c*, has the tips at *d*_e_ + *d*_i_ = 2.69 Å. The pair of scattered points of spikes in the fingerprint plot delineated into H⋯N/N⋯H, Fig. 5 ▸ *d* (19.3%), have the tips at *d*_e_ + *d*_i_ = 1.81 Å. The H⋯O/O⋯H contacts, Fig. 5 ▸ *e* (9.6%), have the tips at *d*_e_ + *d*_i_ = 2.65 Å. The C⋯C contacts, Fig. 5 ▸ *f*, contribute 0.9% to the HS and appear as a pair of scattered points of spikes with the tips at *d*_e_ + *d*_i_ = 3.60 Å. Finally, the C⋯N/N⋯C contacts, Fig. 5 ▸ *g*, make only a 0.5% contribution to the HS and have a low-density distribution of points.

## Density Functional Theory calculations   

**Figure 6:**
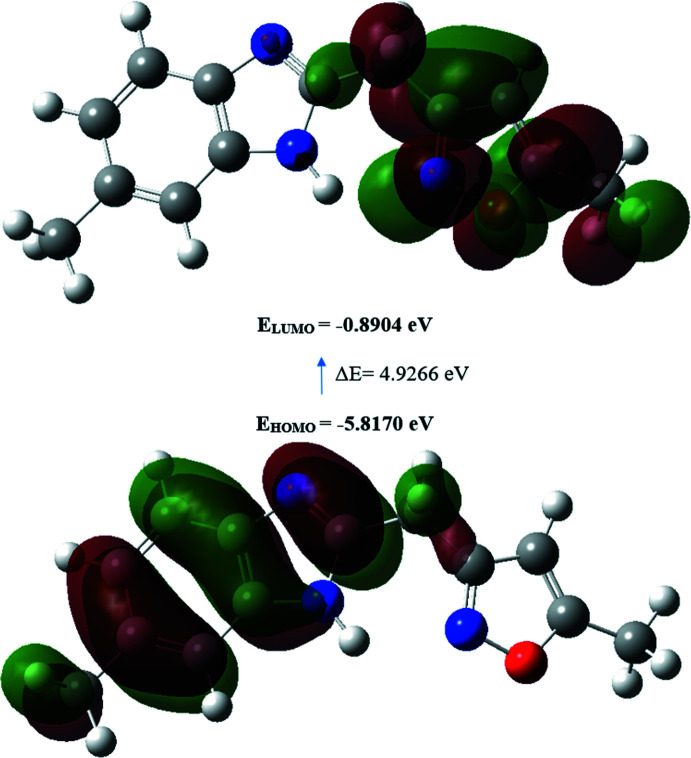
The energy band gap of 6-methyl-2-[(5-methyl­isoxazol-3-yl)meth­yl]-1*H*-benzimidazole.

The structure in the gas phase of the title compound was optimized by means of density functional theory. The density functional theory calculation was performed by the hybrid B3LYP method and the 6–311 G(d,p) basis-set, which is based on Becke’s model (Becke, 1993 ▸) and considers a mixture of the exact (Hartree–Fock) and density functional theory exchange utilizing the B3 functional, together with the LYP correlation functional (Lee *et al.*, 1988 ▸). After obtaining the converged geometry, the harmonic vibrational frequencies were calculated at the same theoretical level to confirm that the number of imaginary frequencies is zero for the stationary point. Both the geometry optimization and harmonic vibrational frequency analysis of the title compound were performed with the *GAUSSIAN 09* program (Frisch *et al.*, 2009 ▸). The theoretical and experimental results related to bond lengths and angles are in good agreement, as well as with the results of the previous structural study of 5,6-dimethyl-2-[(5-methyl-1,2-oxazol-3-yl)meth­yl]-1-(prop-2-en-1-yl)-1*H*-benzimidazole, (III) (Benyahya *et al.*, 2017 ▸) and 5-methyl-3-(1-(2-pyridyl­meth­yl)-1*H*-benzimidazol-2-ylmeth­yl)isoxazole, (IV) (Doumbia *et al.*, 2009 ▸), which are summarized in Table 2 ▸. Calculated numerical values for title compound including electronegativity (*χ*), hardness (*η*), ionization potential (*I*), dipole moment (*μ*), electron affinity (*A*), electrophilicity (*ω*) and softness (*σ*) are collated in Table 3 ▸. The electron transition from the highest occupied mol­ecular orbital (HOMO) to the lowest unoccupied mol­ecular orbital (LUMO) energy level is shown in Fig. 6 ▸. The HOMO and LUMO are localized in the plane extending over the whole 6-methyl-2-[(5-methyl­isoxazol-3-yl)meth­yl]-1*H*-benzimidazole system. The energy band gap [*ΔE* = *E*_LUMO_ - *E*_HOMO_] of the mol­ecule is 4.9266 eV, and the frontier mol­ecular orbital energies, *E*_HOMO_ and *E*_LUMO_, are −5.8170 and −0.8904 eV, respectively.

## Database survey   

**Figure 7:**
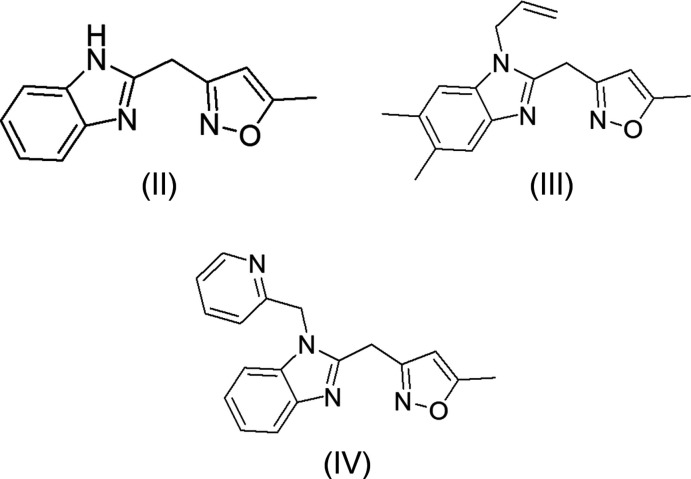
Structural fragments (II), (III) and (IV) used in the database survey.

A search of the Cambridge Structural Database (CSD version 5.40, updated March 2020; Groom *et al.*, 2016 ▸) with the 2-methyl­benzimidazole fragment yielded multiple matches. Of these, three had an isoxazol-3-yl substituent comparable to (I) and they are shown in Fig. 7 ▸. The first compound (II) (refcode REQZIW; Attar *et al.*, 2001 ▸) has no substituent on the phenyl ring. For the second one (III) (refcode FECPIP; Benyahya *et al.*, 2017 ▸) the phenyl ring is disubstituted with an allyl substituent on nitro­gen 1. The third one (IV) (refcode PUGLAF; Doumbia *et al.*, 2009 ▸) carries pyridin-2-ylmethyl on nitro­gen 1. The benzimidazole and isoxazole moieties are planar and make a dihedral angle of 76,15 (4)° in REQZIW. In FECPIP, the benzimidazole moiety is slightly non-planar, as indicated by the dihedral angle of 1.3 (1)° between the five- and six-membered rings. The isoxazole ring is planar to within 0.005 (1) Å and makes a dihedral angle of 89.78 (8)° with the benzimidazole ring. On the other hand, in PUGLAF, the fused-ring system is essentially planar, with a maximum deviation of 0.019 (1) Å. It forms inter­planar angles of 70.03 (7)° with the isoxazole ring and 81.68 (7)° with the pyridine ring. The two latter rings are also planar, the maximum deviations from the mean planes being 0.0028 (15) and 0.0047 (12) Å. In (I), The isoxazole ring is inclined to the mean plane of the benzimidazole ring by 69.28 (14)° which is approximately the same as in PUGLAF, but less tilted than in REQZIW and FECPIP.

## Synthesis and crystallization   

(Z)-7-Methyl-4-(2-oxo­propyl­idene)-1,5-benzodiazepin-2-one (2.3 g, 0.01 mol) and hydroxyl­amine hydro­chloride (0.7 g, 0.01 mol) were brought to reflux in 40 ml of methanol for 2 h. After neutralization with NaHCO_3_, the compound that precipitated was filtered and recrystallized from ethyl acetate. The product was dissolved to saturation in ethyl acetate and crystals were obtained by evaporation at room temperature. yield: 70%; m.p. 465–467 K; IR [KBr, γ(cm^−1^)]: γ_NH_ = 3416; γ_CH_ = 3012–3263; γ_C=N–C=C_= 1525–1672; ^1^H NMR [300MHz, DMSO-*d*_6_, δ(ppm)]: 2.32 (*s*, 3H, CH_3_ isoxazole); 2.57 (*s*, 3H, CH_3_ benzimidazole); 4.23 (*s*, 2H, CH_2_); 6.22 (*s*, 1H, CH isoxazole); 7.00–7.60 (*m*, 3H, CH_ar_); 5.0 (*s*, 1H, NH). ^13^C NMR [75MHz, DMSO-*d*_6_, δ(ppm)]: 13.2 (CH_3_ isoxazole); 24.3 (CH_3_ benzimidazole); 26.7 (CH_2_); 101.8 (CH isoxazole); 115.2–125.8 (CH ar­yl); 132.7–169.6 (C quaternary).

## Refinement   

Crystal data, data collection and structure details refinement are given in Table 4 ▸. Hydrogen atoms were located in the first difference-Fourier map. C-bound H atoms were positioned geometrically (C—H = 0.93–0.97 Å) and included as riding contributions with *U*_iso_(H) = 1.2*U*_eq_(C) (1.5 for methyl groups). At the end of the refinement, the final difference Fourier map showed no residual peaks of chemical significance.

## Supplementary Material

Crystal structure: contains datablock(s) I. DOI: 10.1107/S2056989021002723/tx2037sup1.cif

Structure factors: contains datablock(s) I. DOI: 10.1107/S2056989021002723/tx2037Isup2.hkl

CCDC reference: 2048487

Additional supporting information:  crystallographic information; 3D view; checkCIF report

## Figures and Tables

**Table 1:** Hydrogen-bond geometry (Å, °)

*D*—H⋯*A*	*D*—H	H⋯*A*	*D*⋯*A*	*D*—H⋯*A*
N8—H8⋯N15^i^	0.89 (3)	1.96 (3)	2.830 (3)	167 (2)
C4—H4⋯N2^ii^	0.93	2.57	3.447 (3)	157

Symmetry codes: (i) x-{\script{1\over 2}}, y, -z+{\script{3\over 2}}; (ii) -x+{\script{3\over 2}}, y+{\script{1\over 2}}, z.

**Table 2:** Comparison of selected (X-ray and DFT bond lengths and angles (Å, °) in the title compound and related structures

	X-ray	B3LYP/6–311G(d,p)	(III)*^*a*^*	(IV)*^*b*^*
O1—N2	1.413 (3)	1.3949	1.417	1.4100
O1—C5	1.339 (4)	1.3481	1.356	1.3526
N2—C3	1.299 (3)	1.3115	1.304	1.3044
C3—C6	1.488 (4)	1.5065	1.501	1.504
C5—C17	1.485 (4)	1.4868	1.476	1.478
C6—C7	1.488 (4)	1.5026	1.498	1.494
C7—N8	1.349 (3)	1.3755	1.377	1.3720
C7—N15	1.320 (3)	1.3092	1.312	1.3079
N8—C9	1.371 (3)	1.3814	1.386	1.3840
C11—C16	1.500 (4)	1.5112	1.504	–
C14—N15	1.391 (3)	1.388	1.400	1.3880
				
C5—O1—N2	108.2 (2)	109.1398	108.37	108.57
C3—N2—O1	105.5 (2)	106.0707	105.15	105.28
N2—C3—C4	111.3 (2)	111.0906	112.00	111.51
N2—C3—C6	118.9 (2)	120.8172	120.16	119.88
O1—C5—C17	117.0 (3)	116.8621	116.33	115.90
C4—C5—O1	109.5 (3)	109.3513	109.34	109.15
N8—C7—C6	121.7 (2)	122.8089	123.02	122.62
N15—C7—C6	125.6 (2)	123.8733	123.28	124.10
N15—C7—N8	112.7 (2)	113.2373	113.69	113.28
C7—N8—C9	107.59 (19)	106.9514	106.09	106.49
N8—C9—C14	105.29 (19)	104.6015	105.63	105.05
C13—C14—N15	130.8 (2)	130.4265	129.98	129.63
N15—C14—C9	109.67 (19)	110.2891	110.23	110.42
C7—N15—C14	104.72 (19)	104.9141	104.36	104.75

Notes: (*a*) Results of the previous DFT-optimized geometry of 5,6-dimethyl-2-[(5-methyl-1,2-oxazol-3-yl)meth­yl]-1-(prop-2-en-1-yl)-1*H*-benzimidazole (Benyahya *et al.*, 2017 ▸); (*b*) results of the previous crystallographic study of 5-methyl-3-(1-(2-pyridyl­meth­yl)-1*H*-benzimidazol-2-ylmeth­yl)isoxazole (Doumbia *et al.*, 2009 ▸)

**Table 3:** Calculated energies

Mol­ecular Energy	Title Compound
Total Energy *TE* (eV)	−20214.1624
*E*_HOMO_ (eV)	−5.8170
*E*_LUMO_ (eV)	−0.8904
Gap, *ΔE* (eV)	4.9266
Dipole moment, *μ* (Debye)	4.4403
Ionization potential, *I* (eV)	5.8170
Electron affinity, *A*	0.8904
Electronegativity, *χ*	3.3537
Hardness, *η*	2.4633
Electrophilicity, index *ω*	2.2830
Softness, *σ*	0.4060
Fraction of electron transferred, *ΔN*	0.7401

**Table 4:** Experimental details

Crystal data	
Chemical formula	C_13_H_13_N_3_O
*M*_r_	227.26
Crystal system, space group	Orthorhombic, *P* *b* *c* *a*
Temperature (K)	294
*a*, *b*, *c* (Å)	9.6545 (6), 11.2437 (6), 22.9108 (14)
*V* (Å^3^)	2487.0 (3)
*Z*	8
Radiation type	Mo *K*α
μ (mm^−1^)	0.08
Crystal size (mm)	0.35 × 0.2 × 0.2

Data collection	
Diffractometer	Rigaku Oxford Diffraction SuperNova, Single source at offset/far, Eos
Absorption correction	Multi-scan (*CrysAlis PRO*; Rigaku OD, 2018 ▸)
*T*_min_, *T*_max_	0.883, 1.000
No. of measured, independent and observed [*I* > 2σ(*I*)] reflections	13352, 2519, 1723
*R*_int_	0.024
(sin θ/λ)_max_ (Å^−1^)	0.625

Refinement	
*R*[*F* ^2^ > 2σ(*F* ^2^)], *wR*(*F* ^2^), *S*	0.064, 0.203, 1.05
No. of reflections	2519
No. of parameters	160
H-atom treatment	H atoms treated by a mixture of independent and constrained refinement
Δρ_max_, Δρ_min_ (e Å^−3^)	0.33, −0.26

Computer programs: *CrysAlis PRO* (Rigaku OD, 2018 ▸), *SHELXT* (Sheldrick, 2015*a* ▸), *SHELXL2016/4* (Sheldrick, 2015*b* ▸) and *OLEX2* (Dolomanov *et al.*, 2009 ▸).

## supplementary crystallographic information

### Crystal data

**Table:**

C_13_H_13_N_3_O	*D*_x_ = 1.214 Mg m^−^^3^
*M**_r_* = 227.26	Mo *K*α radiation, λ = 0.71073 Å
Orthorhombic, *P**b**c**a*	Cell parameters from 3531 reflections
*a* = 9.6545 (6) Å	θ = 2.9–23.3°
*b* = 11.2437 (6) Å	µ = 0.08 mm^−^^1^
*c* = 22.9108 (14) Å	*T* = 294 K
*V* = 2487.0 (3) Å^3^	Prism, brown
*Z* = 8	0.35 × 0.2 × 0.2 mm
*F*(000) = 960	

### Data collection

**Table:**

Rigaku Oxford Diffraction SuperNova, Single source at offset/far, Eos diffractometer	2519 independent reflections
Radiation source: micro-focus sealed X-ray tube, SuperNova (Mo) X-ray Source	1723 reflections with *I* > 2σ(*I*)
Mirror monochromator	*R*_int_ = 0.024
Detector resolution: 15.9631 pixels mm^-1^	θ_max_ = 26.4°, θ_min_ = 2.8°
ω scans	*h* = −10→12
Absorption correction: multi-scan (CrysAlisPro; Rigaku OD, 2018)	*k* = −14→13
*T*_min_ = 0.883, *T*_max_ = 1.000	*l* = −28→28
13352 measured reflections	

### Refinement

**Table:**

Refinement on *F*^2^	Primary atom site location: dual
Least-squares matrix: full	Hydrogen site location: mixed
*R*[*F*^2^ > 2σ(*F*^2^)] = 0.064	H atoms treated by a mixture of independent and constrained refinement
*wR*(*F*^2^) = 0.203	*w* = 1/[σ^2^(*F*_o_^2^) + (0.0963*P*)^2^ + 0.6658*P*] where *P* = (*F*_o_^2^ + 2*F*_c_^2^)/3
*S* = 1.05	(Δ/σ)_max_ < 0.001
2519 reflections	Δρ_max_ = 0.33 e Å^−^^3^
160 parameters	Δρ_min_ = −0.26 e Å^−^^3^
0 restraints	

### Special details

**Table:**

Geometry. All esds (except the esd in the dihedral angle between two l.s. planes) are estimated using the full covariance matrix. The cell esds are taken into account individually in the estimation of esds in distances, angles and torsion angles; correlations between esds in cell parameters are only used when they are defined by crystal symmetry. An approximate (isotropic) treatment of cell esds is used for estimating esds involving l.s. planes.

### Fractional atomic coordinates and isotropic or equivalent isotropic displacement parameters (Å^2^)

**Table:**

	*x*	*y*	*z*	*U*_iso_*/*U*_eq_	
O1	0.8450 (3)	0.0193 (2)	0.57078 (9)	0.1116 (8)	
N2	0.7873 (3)	−0.0264 (2)	0.62282 (11)	0.0916 (8)	
C3	0.7336 (2)	0.0646 (2)	0.64943 (11)	0.0649 (6)	
C4	0.7542 (3)	0.1693 (2)	0.61779 (12)	0.0852 (9)	
H4	0.726197	0.245615	0.628050	0.102*	
C5	0.8227 (4)	0.1368 (3)	0.56964 (12)	0.0962 (10)	
C6	0.6621 (3)	0.0459 (3)	0.70624 (12)	0.0802 (8)	
H6A	0.562991	0.042286	0.699489	0.096*	
H6B	0.690721	−0.030027	0.722308	0.096*	
C7	0.6914 (2)	0.1410 (2)	0.74970 (10)	0.0628 (6)	
N8	0.5894 (2)	0.19185 (18)	0.78133 (8)	0.0616 (5)	
C9	0.6490 (2)	0.2765 (2)	0.81631 (9)	0.0578 (6)	
C10	0.5943 (3)	0.3565 (2)	0.85619 (10)	0.0727 (7)	
H10	0.499999	0.357606	0.864518	0.087*	
C11	0.6850 (3)	0.4350 (3)	0.88328 (11)	0.0815 (8)	
C12	0.8243 (3)	0.4291 (3)	0.87071 (12)	0.0855 (9)	
H12	0.883742	0.481816	0.889438	0.103*	
C13	0.8799 (3)	0.3491 (3)	0.83190 (11)	0.0785 (8)	
H13	0.974628	0.347148	0.824563	0.094*	
C14	0.7902 (2)	0.2713 (2)	0.80401 (10)	0.0615 (6)	
N15	0.81476 (19)	0.18510 (19)	0.76175 (9)	0.0678 (6)	
C16	0.6344 (5)	0.5270 (4)	0.92565 (15)	0.1269 (13)	
H16A	0.638258	0.604086	0.907763	0.190*	
H16B	0.691872	0.526214	0.959831	0.190*	
H16C	0.540500	0.509387	0.936487	0.190*	
C17	0.8775 (7)	0.2031 (4)	0.51859 (15)	0.174 (2)	
H17A	0.975882	0.191938	0.516094	0.261*	
H17B	0.857462	0.286270	0.523070	0.261*	
H17C	0.834513	0.174111	0.483589	0.261*	
H8	0.501 (3)	0.182 (2)	0.7723 (11)	0.079 (8)*	

### Atomic displacement parameters (Å^2^)

**Table:**

	*U*^11^	*U*^22^	*U*^33^	*U*^12^	*U*^13^	*U*^23^
O1	0.153 (2)	0.0868 (15)	0.0948 (15)	−0.0016 (13)	0.0324 (14)	−0.0116 (12)
N2	0.118 (2)	0.0609 (13)	0.0961 (16)	0.0068 (12)	0.0172 (14)	0.0016 (12)
C3	0.0657 (15)	0.0504 (12)	0.0785 (15)	−0.0017 (10)	−0.0074 (12)	−0.0070 (11)
C4	0.121 (2)	0.0538 (14)	0.0809 (18)	−0.0009 (14)	−0.0090 (17)	−0.0022 (12)
C5	0.150 (3)	0.0717 (18)	0.0673 (17)	−0.0246 (18)	−0.0082 (17)	−0.0019 (14)
C6	0.0720 (17)	0.0801 (18)	0.0885 (18)	−0.0208 (13)	0.0074 (13)	−0.0092 (14)
C7	0.0463 (13)	0.0698 (14)	0.0724 (14)	−0.0068 (11)	0.0015 (10)	0.0054 (11)
N8	0.0404 (11)	0.0732 (13)	0.0711 (12)	−0.0031 (9)	−0.0037 (9)	0.0051 (10)
C9	0.0494 (12)	0.0671 (14)	0.0569 (12)	0.0038 (10)	−0.0035 (10)	0.0105 (10)
C10	0.0704 (16)	0.0799 (17)	0.0678 (14)	0.0137 (13)	−0.0001 (12)	0.0093 (13)
C11	0.103 (2)	0.0806 (18)	0.0608 (15)	0.0105 (16)	−0.0089 (14)	0.0014 (12)
C12	0.094 (2)	0.093 (2)	0.0687 (16)	−0.0185 (16)	−0.0220 (15)	0.0013 (14)
C13	0.0649 (16)	0.099 (2)	0.0717 (15)	−0.0169 (14)	−0.0119 (13)	0.0017 (15)
C14	0.0528 (13)	0.0738 (15)	0.0580 (12)	−0.0059 (10)	−0.0080 (10)	0.0101 (11)
N15	0.0453 (11)	0.0817 (14)	0.0763 (13)	−0.0062 (9)	0.0034 (9)	−0.0031 (10)
C16	0.157 (3)	0.126 (3)	0.097 (2)	0.025 (3)	−0.003 (2)	−0.029 (2)
C17	0.311 (7)	0.140 (3)	0.072 (2)	−0.080 (4)	0.008 (3)	0.011 (2)

### Geometric parameters (Å, º)

**Table:**

O1—N2	1.413 (3)	C9—C14	1.393 (3)
O1—C5	1.339 (4)	C10—H10	0.9300
N2—C3	1.299 (3)	C10—C11	1.390 (4)
C3—C4	1.396 (4)	C11—C12	1.377 (4)
C3—C6	1.488 (4)	C11—C16	1.500 (4)
C4—H4	0.9300	C12—H12	0.9300
C4—C5	1.337 (4)	C12—C13	1.374 (4)
C5—C17	1.485 (4)	C13—H13	0.9300
C6—H6A	0.9700	C13—C14	1.386 (3)
C6—H6B	0.9700	C14—N15	1.391 (3)
C6—C7	1.488 (4)	C16—H16A	0.9600
C7—N8	1.349 (3)	C16—H16B	0.9600
C7—N15	1.320 (3)	C16—H16C	0.9600
N8—C9	1.371 (3)	C17—H17A	0.9600
N8—H8	0.88 (3)	C17—H17B	0.9600
C9—C10	1.387 (3)	C17—H17C	0.9600
			
C5—O1—N2	108.2 (2)	C9—C10—C11	117.8 (3)
C3—N2—O1	105.5 (2)	C11—C10—H10	121.1
N2—C3—C4	111.3 (2)	C10—C11—C16	121.4 (3)
N2—C3—C6	118.9 (2)	C12—C11—C10	119.4 (3)
C4—C3—C6	129.7 (2)	C12—C11—C16	119.2 (3)
C3—C4—H4	127.2	C11—C12—H12	118.4
C5—C4—C3	105.6 (3)	C13—C12—C11	123.3 (3)
C5—C4—H4	127.2	C13—C12—H12	118.4
O1—C5—C17	117.0 (3)	C12—C13—H13	121.1
C4—C5—O1	109.5 (3)	C12—C13—C14	117.8 (3)
C4—C5—C17	133.5 (3)	C14—C13—H13	121.1
C3—C6—H6A	108.9	C13—C14—C9	119.5 (2)
C3—C6—H6B	108.9	C13—C14—N15	130.8 (2)
H6A—C6—H6B	107.7	N15—C14—C9	109.67 (19)
C7—C6—C3	113.3 (2)	C7—N15—C14	104.72 (19)
C7—C6—H6A	108.9	C11—C16—H16A	109.5
C7—C6—H6B	108.9	C11—C16—H16B	109.5
N8—C7—C6	121.7 (2)	C11—C16—H16C	109.5
N15—C7—C6	125.6 (2)	H16A—C16—H16B	109.5
N15—C7—N8	112.7 (2)	H16A—C16—H16C	109.5
C7—N8—C9	107.59 (19)	H16B—C16—H16C	109.5
C7—N8—H8	121.4 (17)	C5—C17—H17A	109.5
C9—N8—H8	129.2 (17)	C5—C17—H17B	109.5
N8—C9—C10	132.5 (2)	C5—C17—H17C	109.5
N8—C9—C14	105.29 (19)	H17A—C17—H17B	109.5
C10—C9—C14	122.2 (2)	H17A—C17—H17C	109.5
C9—C10—H10	121.1	H17B—C17—H17C	109.5
			
O1—N2—C3—C4	−0.7 (3)	N8—C7—N15—C14	−0.4 (3)
O1—N2—C3—C6	179.3 (2)	N8—C9—C10—C11	−177.5 (2)
N2—O1—C5—C4	0.0 (4)	N8—C9—C14—C13	178.6 (2)
N2—O1—C5—C17	179.1 (3)	N8—C9—C14—N15	0.5 (2)
N2—C3—C4—C5	0.7 (3)	C9—C10—C11—C12	−1.3 (4)
N2—C3—C6—C7	139.7 (3)	C9—C10—C11—C16	178.2 (3)
C3—C4—C5—O1	−0.4 (4)	C9—C14—N15—C7	−0.1 (3)
C3—C4—C5—C17	−179.3 (4)	C10—C9—C14—C13	−0.6 (3)
C3—C6—C7—N8	133.7 (2)	C10—C9—C14—N15	−178.7 (2)
C3—C6—C7—N15	−46.0 (4)	C10—C11—C12—C13	0.5 (4)
C4—C3—C6—C7	−40.4 (4)	C11—C12—C13—C14	0.3 (4)
C5—O1—N2—C3	0.4 (3)	C12—C13—C14—C9	−0.3 (4)
C6—C3—C4—C5	−179.3 (3)	C12—C13—C14—N15	177.4 (2)
C6—C7—N8—C9	−179.1 (2)	C13—C14—N15—C7	−177.9 (3)
C6—C7—N15—C14	179.3 (2)	C14—C9—C10—C11	1.4 (3)
C7—N8—C9—C10	178.4 (2)	N15—C7—N8—C9	0.7 (3)
C7—N8—C9—C14	−0.7 (2)	C16—C11—C12—C13	−179.0 (3)

### Hydrogen-bond geometry (Å, º)

**Table:**

*D*—H···*A*	*D*—H	H···*A*	*D*···*A*	*D*—H···*A*
N8—H8···N15^i^	0.89 (3)	1.96 (3)	2.830 (3)	167 (2)
C4—H4···N2^ii^	0.93	2.57	3.447 (3)	157

Symmetry codes: (i) *x*−1/2, *y*, −*z*+3/2; (ii) −*x*+3/2, *y*+1/2, *z*.
